# Supplementary material for: HLA-E and Its Soluble Form as Indicators of a Sex-Specific Immune Response in Patients with Oral Squamous Cell Carcinoma
Source: Int J Mol Sci. 2023 Nov 24;24(23):16699. doi: 10.3390/ijms242316699 (PMC10706335; doi:10.3390/ijms242316699)
Supplement: Supplementary file 1 [file ijms-24-16699-s001.zip › ijms-2707947-supplementary.pdf]

# HLA-E and its soluble form as indicators of a sex-specific immune response in patients with Oral Squamous Cell Carcinoma

**Anne Radermacher, Michael Fehrenz, Tamara Bellin, Carolina Claßen, Laura Möller, Ann-Kristin Struckmeier, Mathias Wagner, Philipp Wartenberg, Julius Moratin, Christian Freudlsperger, Kolja Freier, Dominik Horn**

**Table S1**: Descriptive statistics on age distribution of tumor group of the ELISA collective

| Characteristics | N | Minimum | Maximum | Mean | SD |
|---|---|---|---|---|---|
| Age | 29 | 48 | 93 | 65.79 | 12.219 |

**Table S2:** Descriptive statistics on age distribution of control group of the ELISA collective

| Characteristics | N | Minimum | Maximum | Mean | SD |
|---|---|---|---|---|---|
| Age | 41 | 20 | 86 | 59 | 15.1 |

**Table S3:** Kolmogorov-Smirnov and Shapiro-Wilk-test of normal distribution of ELISA group

| | Kolmogorov-Smirnov | | | Shapiro-Wilk | | |
|---|---|---|---|---|---|---|
| | **Statistics** | **Df** | **Significance** | **Statistics** | **Df** | **Significance** |
| sHLA-E-OD | 0.12 | 74 | 0.020 | 0.923 | 74 | <0.001 |

**Table S4:** Spearman´s correlation of the clinicopathological parameters sex, age, N-classification and UICC-Stage with sHLA-E levels of the ELISA collective

| Characteristics | Spearman´s Rho | p-value |
|---|---|---|
| Sex | 0.357 | 0.045* |
| Age | 0.202 | 0.294 |
| T-classification | 0.068 | 0.711 |
| N-classification | -0.115 | 0.546 |
| UICC-Stage | 0.031 | 0.869 |

Abbreviations: UICC= Union Internationale Contre le Cancer, *= p-value < 0.05

**Table S5: Mann**-Whitney-U-Test. Compares mean ranks of sHLA-E-levels of the tumor group between male and female patients

| Factor | Groups compared | N | Mean Rank | Mann-Whitney-U | W | p-value (exact) |
|---|---|---|---|---|---|---|
| sHLA-E concentration | Men OSCC | 18 | 13.64 | 177.5 | 282.5 | 0.049* |
| | Women OSCC | 14 | 20.18 | | | |

*= p-value < 0.05

**Table S6**: Mann-Whitney-U-Test. Compares mean ranks of sHLA-E-levels of the control group between male and female patients

| Factor | Groups compared | N | Mean Rank | Mann-Whitney-U | W | p-value (exact) |
|---|---|---|---|---|---|---|
| sHLA-E concentration | Men Control group | 22 | 22.48 | 198.5 | 408.5 | 0.587 |
| | Women Tumor group | 20 | 20.43 | | | |

**Table S7:** Mann-Whitney-U-Test. Compares mean ranks of sHLA-E-levels of the tumor group and the control group

| Factor | Groups compared | N | Mean Rank | Mann-Whitney-U | W | p-value (exact) |
|---|---|---|---|---|---|---|
| sHLA-E concentration | Tumor group | 32 | 30.91 | 461 | 989 | 0.021* |
| | Control group | 42 | 40.52 | | | |

*= p-value < 0.05

**Table S8:** Mann-Whitney-U-Test. Compares mean sHLA-E-levels of males of tumor group and males of control group

| Factor | Groups compared | N | Mean Rank | Mann-Whitney-U | W | p-value (exact) |
|---|---|---|---|---|---|---|
| sHLA-E concentration | Men Tumor group | 18 | 14.14 | 312.5 | 565.5 | 0.001* |
| | Men Control group | 22 | 25.7 | | | |

*= p-value < 0.05

**Table S9:** Mann-Whitney-U-Test. Compares mean sHLA-E-levels of females of tumor group and males of control group

| Factor | Groups compared | N | Mean Rank | Mann-Whitney-U | W | p-value (exact) |
|---|---|---|---|---|---|---|
| sHLA-E concentration | Women Tumor group | 14 | 17.29 | 134 | 353 | 0.916 |
| | Women Control group | 20 | 17.65 | | | |
